# Cellular Origin(s) of Congenital Diaphragmatic Hernia

**DOI:** 10.3389/fped.2021.804496

**Published:** 2021-11-30

**Authors:** Gabriëla G. Edel, Gerben Schaaf, Rene M. H. Wijnen, Dick Tibboel, Gabrielle Kardon, Robbert J. Rottier

**Affiliations:** ^1^Department of Pediatric Surgery and Intensive Care, Erasmus MC-Sophia Children's Hospital, Rotterdam, Netherlands; ^2^Department of Cell Biology, Erasmus MC, Rotterdam, Netherlands; ^3^Department of Clinical Genetics, Erasmus MC, Rotterdam, Netherlands; ^4^Department of Pediatrics, Erasmus MC-Sophia Children's Hospital, Rotterdam, Netherlands; ^5^Center for Lysosomal and Metabolic Diseases, Erasmus MC, Rotterdam, Netherlands; ^6^Department of Human Genetics, University of Utah, Salt Lake City, UT, United States

**Keywords:** congenital diaphragmatic hernia (CDH), diaphragm, pleuroperitoneal folds, perivascular cells, mesothelium

## Abstract

Congenital diaphragmatic hernia (CDH) is a structural birth defect characterized by a diaphragmatic defect, lung hypoplasia and structural vascular defects. In spite of recent developments, the pathogenesis of CDH is still poorly understood. CDH is a complex congenital disorder with multifactorial etiology consisting of genetic, cellular and mechanical factors. This review explores the cellular origin of CDH pathogenesis in the diaphragm and lungs and describes recent developments in basic and translational CDH research.

## Introduction

In the past 25 years, the general mortality rate of CDH has decreased to approximately 25%, but the mortality rate remained 50% in patients who receive extracorporeal membrane oxygenation (ECMO) (1, 2). In a recent study, CDH patients were categorized pre-ECMO in a low-risk, moderate-risk or high-risk cohort by their risk score (RS) for mortality, which is based on multiple risk factors, like location of the hernia and weight before ECMO (1). Change in individual likelihood of death overtime was different for each cohort: it was increased in the low risk group, decreased in the moderate risk group and unchanged in the high-risk group. Although the average survival of CDH patients increased in the past decades as a result of advancements in prenatal diagnosis, CDH pathogenesis remains still poorly understood. The main reason is that CDH is a complex congenital disorder with multifactorial etiology including genetic, cellular and environmental factors (3–5). In this review, we will discuss different tissues and cell types that are implicated in CDH pathogenesis and review recent developments in basic and translational research in CDH.

## Diaphragm/PPF

The diaphragm is an essential muscle that is critical for proper respiration and forms a barrier between the thoracic and abdominal cavities (6). The diaphragm develops from multiple embryonic sources. Of primary importance are the pleuroperitoneal folds (PPFs). The PPFs are paired transient pyramidal-shaped structures located between the thoracic (pleural) and abdominal (peritoneal) cavities. The PPFs expand dorsally and ventrally across the cranial surface of the liver to give rise to the diaphragm's muscle connective tissue and central tendon (7–9). The somites, segmental structures lying adjacent to the neural tube, are the source of the diaphragm muscle. Muscle progenitors emigrate from the cervical somites to the nascent PPFs and as the PPFs expand, the progenitors migrate and fuse into the radial array of costal myofibers (7–11). In addition, the diaphragm is innervated by nerves that arise from the C3-C5 segments of the neural tube and it is vascularized by endothelial cells derived from the somites and likely splanchnic lateral plate mesoderm (8–10).

The PPFs are essential for the morphogenesis of the diaphragm and defects in the PPFs lead to CDH. Experiments in mice genetically tracking the development of the PPFs have established that the expansion of the PPFs drives the overall morphogenesis of the diaphragm and guide diaphragm muscle development (7). Defects in the development of the PPFs are a significant source of CDH. Most CDH-implicated genes that have been examined are expressed in the PPF cells (7, 12–15). Mutations in CDH-implicated genes can lead to the incomplete expansion of the PPFs and thus lead to incompletely developed diaphragms that allow herniation of abdominal contents into thoracic cavity [e.g., (16)]. Alternatively, PPFs harboring mutations may be unable to signal to muscle progenitors [e.g., (7)], leading to defective progenitor migration to the PPFs, increased apoptosis, and/or decreased proliferation or differentiation into myofibers. In such scenarios, defects in the PPF cells lead to cell non-autonomous effects on neighboring muscle, resulting in weaker muscleless regions that allow herniation (7). To date, all evidence suggests that CDH arises from primary defects in the PPFs by either defective generation/migration of cell populations or impaired muscularization of the diaphragm, with little evidence to support a primary role for defects in muscle cells.

## Lung Mesothelium

Pulmonary hypoplasia is another characteristic of CDH. Although a lower amount of alveolar type I cells have been identified in nitrofen-induced CDH (17, 18), the tissue that is primarily defective in CDH-associated hypoplasia is not yet clear. A potential cellular source in CDH pathogenesis is the lung mesothelium, which contributes to different cell populations in the lung and is important for proper mesenchymal growth. The pleural mesothelium is a monolayer of cells that forms a lining around the lungs and is derived from the embryonic mesoderm (19). Although mesothelial cells are mesenchymal in origin, they have epithelial characteristics (20). During development, pulmonary mesothelial cells (PMCs) undergo epithelial-mesenchymal transition and differentiate to contribute to different cell populations in the pulmonary mesenchyme under the influence of active hedgehog signaling (21).

Several studies reported conflicting data to what extent the embryonic mesothelium contributes to the pulmonary mesenchyme. These studies employed different *Wilm's tumor 1* (*Wt1*)-dependent driver lines, which is a gene encoding a zinc finger transcription factor expressed in mesothelial cells and diaphragm and that is associated with CDH (22). *Wt1*-dependent lines were used to trace WT1^+^ mesothelial cells and analyzed the fate of the progeny at several time points, which most likely explains the variation in their results. Que et al. (23) reported that WT1^+^ lung mesothelial cells contribute to vascular smooth muscle cells (SMCs) and to alveoli, which were potentially interstitial fibroblasts, alveolar myofibroblasts or endothelial cells. Dixit et al. chose a conditional strategy to lineage trace *Wt1*-expressing mesothelial cells and reported contribution of PMCs to vascular and bronchial smooth muscle cells, as well as fibroblasts (21). Cano et al. (24) focused on the contribution of PMCs to different lung cell types during embryonic lung development and observed contribution to a wider range of cell types including endothelial cells, airway and vascular smooth muscle cells, pulmonary cartilage and fibroblasts. Lastly, Von Gise et al. (25) found that labeling WT1^+^ cells at E10.5 resulted in a small contribution to bronchial- and vascular smooth muscle cells, while the majority differentiated into PDGFRα^+^-fibroblasts or PDGFRβ^+^/NG2^+^-pericytes. Von Gise et al. (25) also showed that only fetal and not postnatal PMCs are capable of differentiating into pulmonary mesenchymal cell types. Postnatal PMCs remain in the mesothelial lining and do not migrate out or differentiate into other cell types. As such, postnatal PMCs do not seem to contribute to the lung parenchyma during normal lung homeostasis or after injury (25). In conclusion, mesothelial cells contribute to the bronchial- and vascular smooth muscle cell population as well as to fibroblasts and pericytes and that this only occurs during embryonic development and not postnatally (21, 25).

Besides being a progenitor source during lung development, the mesothelium also acts as a signaling source. One important pathway during development of the lungs is the FGF signaling pathway, in which FGF9 and FGF10 are essential (26). FGF10 is expressed in the lung mesenchyme, while FGF9 is expressed in the mesothelium and epithelium, signaling to the submesothelial- and subepithelial mesenchyme, respectively (27, 28). Mesothelial- and epithelial-produced FGFs have a different function: mesothelial-derived FGF9 is mainly responsible for mesenchymal growth by maintaining mesenchymal FGF-WNT/β-catenin signaling, whereas epithelial-derived FGF9 influences epithelial branching (29). One study reported reduced pulmonary FGF9 expression in the nitrofen-induced CDH rat model (30). However, recombinant co-cultures of fibroblasts and epithelial cells of nitrofen-treated- and control rats showed that not epithelial cells, but fibroblasts, are defective in nitrofen-induced hypoplastic lungs, showing decreased apoptosis and increased proliferation (31). Since the mesothelium is a source for mesenchymal fibroblasts (21, 23–25) and expresses FGF9 for the regulation of mesenchymal growth (29), the mesothelium is a potential source for CDH-associated lung hypoplasia.

Cano et al. (24) showed that homozygous *Wt1* knock-out (*Wt1*^−/−^) mice had a CDH-like phenotype, abnormally fused lung lobes and reduced immunoreactivity for FGF9 in the pulmonary mesenchyme and mesothelium. A recent study reported similar findings and also reported an aberrant lung branching architecture already before closure of the diaphragm, when WT1 is expressed (32). When *Wt1*^−/−^ lungs were cultured *ex vivo*, lung branching was normal and any hypoplasia that had originated *in vivo*, was restored within 24 h *ex viv*o (32). Additional analyses showed that the space in the chest cavity—that is usually present for the lungs to grow—was nearly absent, explaining why culturing the lungs *ex vivo* without physical constraints recovered branching (32). Aberrant WT1 expression in the lung mesothelium results in defective lung development and CDH as result of limited space in the chest cavity and potentially by defective signaling and migration of mesothelial cells.

In summary, the mesothelium acts as a progenitor source and signaling center for the pulmonary mesenchyme to facilitate proper mesenchymal growth and cellular differentiation. These results associate the lung mesothelium as a cellular contributor to CDH.

## (Peri)Vascular Cells

Besides a diaphragmatic defect and pulmonary hypoplasia, almost all CDH patients have pulmonary hypertension (33, 34), which is caused by an altered development of the pulmonary vasculature and pulmonary vascular remodeling (35). Changes in cell phenotypes, cellular proliferation and defective cell-cell communication have been proposed as underlying causes.

Previously, it was shown that CDH patients have higher abundance of contractile vascular SMCs, which were also more distributed along the proximo-distal axis of the lung vasculature (36). Although inhaled nitric oxide treatment is a successful treatment for preterm babies, it is only effective in a small number of CDH patients and even only beneficial in certain subsets of CDH patients (37). The pathological changes in vascular SMCs indicate a disturbed pulmonary vascular development and might explain the ineffectiveness of inhaled nitric oxide treatment in CDH patients. Another study by Acker et al. (38) showed increased proliferation of pulmonary arterial SMCs and pulmonary arterial SMC hyperplasia in a surgical CDH lamb model. This was not caused by an altered SMC phenotype, but by a disturbed interaction with pulmonary arterial endothelial cells (PAECs), indicating that defective endothelial signaling contributed to SMC hyperplasia and may therefore result in pulmonary hypertension (38). In the nitrofen-induced CDH mouse model, Kool et al. (39) observed an increased pericyte coverage in the large pulmonary vessels and pericytes had a more contractile phenotype. Furthermore, the basement membrane around the midsized vessels was discontinuous, indicating defective cross-talk between pericytes and endothelial cells (39). The impaired cross-talk between those two cell types and the altered pericyte phenotype may be the origin of pulmonary hypertension in CDH.

CDH patients show a decreased vascular growth that contributes to poor disease outcomes. Acker et al. (40) showed in the surgical CDH lamb model reduced proliferation and tube formation capacity of PAECs. They also found a marked reduction in high-proliferative PAECs, which is a progenitor subpopulation of endothelial cells (41, 42). A reduced capillary network was also observed by Kool et al. (39) in the nitrofen-induced CDH mouse model. These results suggest that reduced proliferation of endothelial cells contributes to the decreased vascular growth in CDH patients.

Altogether, several cell types may disturb the lung vascular network, leading to CDH-associated phenotypes. Abnormal phenotypes of endothelial cells, SMCs and pericytes and defective interactions between them may be the basis for the simplified vascular network and pulmonary hypertension in CDH.

## Cellular Models in CDH Research

CDH cannot be attributed to one single source or defect, which makes it hard to study its pathogenesis, but new cell culture models can aid in improving insights at the cellular level. Recently, a cell culture model was described where PPFs from mice were isolated and cultured to outgrow and expand PPF fibroblasts, that maintained expression of key diaphragm genes (43). Pharmacological inhibition or genetic manipulation that causes CDH resulted in reduced *in vitro* proliferation in PPF-derived fibroblasts (43). Also, lung organoids were recently derived from induced pluripotent stem cells (iPSC) from fetuses and infants with CDH and showed reduced generation of lung progenitor cells and impaired epithelial- and mesenchymal differentiation (44). Recently, it was shown that organoid cultures can be obtained with a low input material from clinical samples, like tracheal aspirates from preterm newborns, and this method could also be used to grow organoids from CDH patients without the need of *in vitro* differentiation from iPSCs (45). Furthermore, endothelial cell culture models could aid in understanding defective endothelial cell function (40) and endothelial cell-SMC interaction (38). A differentiation protocol from human iPSCs to endothelial progenitor cells that mimics *in vivo* embryonic vascular development was recently published (42). This method can be used to generate iPSC-derived endothelial cells from CDH patients to eventually use these cells in cell co-culture systems, like organoid cultures. Although CDH is a multifactorial disease, *in vivo* experiments are limited and these kind of culture models will help to understand the pathology of CDH by specific cells, the interaction between different cell types and the molecular mechanisms in patient specific cultures.

## Discussion

In this review, we discussed cellular origins that are associated with CDH pathogenesis, including the diaphragm and PPF, lung mesothelium and (peri)vascular cells. All can contribute to a CDH phenotype, but also extrinsic factors play a role. Because pulmonary defects and alterations in the space of the chest cavity occurred prior to diaphragm closure and may even be the cause of a diaphragm defect (32), it is interesting to study the space in the chest cavity in other CDH models and potentially in human fetuses to serve as an early predictor for CDH. To mimic limited chest space and compression, lung organoids have been subjected to mechanical pressure, which altered their development (44). These results show that extrinsic factors play a significant role in CDH pathogenesis and the influence of limited space and compression should be studied further, since *Wt1* knock-out lungs still had the capacity to develop normally *ex vivo*, which is promising for potential treatment strategies (32).

Intrapleural delivery of compounds has been suggested to target PMCs in idiopathic pulmonary fibrosis, since this may result in increased efficacy combined with reduced systemic toxicity of therapeutic agents (19, 46). This delivery strategy may also be interesting for targeting PMCs during lung development. Mesothelial mobilization is not only implicated in development of the lungs, but also in other organs, like the liver, heart and reproductive system (47). However, it has been shown that this process can differ between different organs and that mesothelial migration in developing lungs differs in timing and pathway dependency compared to other organs, like the heart (48). These features could be used for organ-specific targeting and makes the embryonic mesothelium an interesting therapeutic target.

In summary, defective development, communication or migration of cells from the diaphragm, the mesothelium and (peri)vascular cells of the lungs play an important role in CDH pathogenesis. Improved *in vitro* cell culture models and the generation of patient-specific cultures will provide more insights in cellular and molecular mechanisms that underlie these defects and their use may be beneficial for identification and testing of putative therapeutic agents.

## Author Contributions

GE and GK wrote the manuscript. GS, RR, and DT carefully edited and revised the various versions of the manuscript and approved the final manuscript. RW revised and approved the final manuscript. All authors approved the final manuscript as submitted.

## Funding

This work was supported by the Sophia Foundation for Scientific Research [Grant Nos. S17-20 (GE and RR)].

## Conflict of Interest

The authors declare that the research was conducted in the absence of any commercial or financial relationships that could be construed as a potential conflict of interest.

## Publisher's Note

All claims expressed in this article are solely those of the authors and do not necessarily represent those of their affiliated organizations, or those of the publisher, the editors and the reviewers. Any product that may be evaluated in this article, or claim that may be made by its manufacturer, is not guaranteed or endorsed by the publisher.

## References

[1] Guner YS Delaplain PT Zhang L Di Nardo M Brogan TV Chen Y. Trends in mortality and risk characteristics of congenital diaphragmatic hernia treated with extracorporeal membrane oxygenation. ASAIO J. (2019) 65:509–15. 10.1097/MAT.0000000000000834 29863628 PMC6251767

[2] Gupta VS Harting MT Lally PA Miller CC Hirschl RB Davis CF. Has Survival improved for congenital diaphragmatic hernia? A 25-year review of over 5000 patients from the CDH study group. Pediatrics. (2021) 147(3 MeetingAbstract):939. 10.1542/peds.147.3_MeetingAbstract.939

[3] Schulz F Jenetzky E Zwink N Bendixen C Kipfmueller F Rafat N. Parental risk factors for congenital diaphragmatic hernia – a large German case-control study. BMC Pediatrics. (2021) 21:278. 10.1186/s12887-021-02748-3 34126946 PMC8201820

[4] Mesas Burgos C Ehrén H Conner P Frenckner B. Maternal risk factors and perinatal characteristics in congenital diaphragmatic hernia: a nationwide population-based study. Fetal Diagnosis and Therapy. (2019) 46:385–91. 10.1159/000497619 30982034

[5] Kardon G Ackerman KG McCulley DJ Shen Y Wynn J Shang L. Congenital diaphragmatic hernias: from genes to mechanisms to therapies. Dis Models Mech. (2017) 10:955–70. 10.1242/dmm.028365 28768736 PMC5560060

[6] Merrell AJ Kardon G. Development of the diaphragm – a skeletal muscle essential for mammalian respiration. Febs J. (2013) 280:4026–35. 10.1111/febs.12274 23586979 PMC3879042

[7] Merrell AJ Ellis BJ Fox ZD Lawson JA Weiss JA Kardon G. Muscle connective tissue controls development of the diaphragm and is a source of congenital diaphragmatic hernias. Nat Genet. (2015) 47:496–504. 10.1038/ng.3250 25807280 PMC4414795

[8] Sefton EM Gallardo M Kardon G. Developmental origin and morphogenesis of the diaphragm, an essential mammalian muscle. Dev Biol. (2018) 440:64–73. 10.1101/278507 29679560 PMC6089379

[9] Babiuk RP Zhang W Clugston R Allan DW Greer JJ. Embryological origins and development of the rat diaphragm. J Comp Neurol. (2003) 455:477–87. 10.1002/cne.10503 12508321

[10] Allan DW Greer JJ. Embryogenesis of the phrenic nerve and diaphragm in the fetal rat. J Comp Neurol. (1997) 382:459–68. 10.1002/(SICI)1096-9861(19970616)382:4&lt;459::AID-CNE3&gt;3.0.CO;2-1 9184993

[11] Dietrich S Abou-Rebyeh F Brohmann H Bladt F Sonnenberg-Riethmacher E Yamaai T. The role of SF/HGF and c-Met in the development of skeletal muscle. Development. (1999) 126:1621–9. 10.1242/dev.126.8.1621 10079225

[12] Ackerman KG Herron BJ Vargas SO Huang H Tevosian SG Kochilas L. Fog2 is required for normal diaphragm and lung development in mice and humans. PLoS Genet. (2005) 1:58–65. 10.1371/journal.pgen.0010010 16103912 PMC1183529

[13] Carmona R Cañete A Cano E Ariza L Muñoz-Chápuli R. Conditional deletion of WT1 in the septum transversum mesenchyme causes congenital diaphragmatic hernia in mice. eLife. (2016) 19:e16009. 10.7554/eLife.16009.013 27642710 PMC5028188

[14] Clugston RD Zhang W Greer JJ. Gene expression in the developing diaphragm: significance for congenital diaphragmatic hernia. Am J Physiol Lung Cell Mol Physiol. (2008) 294:L665–75. 10.1152/ajplung.00027.2008 18263670

[15] Paris ND Coles GL Ackerman KG. Wt1 and beta-catenin cooperatively regulate diaphragm development in the mouse. Dev Biol. (2015) 407:40–56. 10.1016/j.ydbio.2015.08.009 26278035 PMC4641796

[16] Clugston RD Zhang W Alvarez S de Lera AR Greer JJ. Understanding abnormal retinoid signaling as a causative mechanism in congenital diaphragmatic hernia. Am J Respir Cell Mol Biol. (2010) 42:276–85. 10.1165/rcmb.2009-0076OC 19448158

[17] Takayasu H Nakazawa N Montedonico S Sugimoto K Sato H Puri P. Impaired alveolar epithelial cell differentiation in the hypoplastic lung in nitrofen-induced congenital diaphragmatic hernia. Pediatr Surg Int. (2007) 23:405–10. 10.1007/s00383-006-1853-y 17245593

[18] Nguyen TM Jimenez J Rendin LE Müller C Westergren-Thorsson G Deprest J. The proportion of alveolar type 1 cells decreases in murine hypoplastic congenital diaphragmatic hernia lungs. PLoS ONE. (2019) 14:e0214793. 10.1371/journal.pone.0214793 30995255 PMC6469843

[19] Batra H Antony VB. The pleura mesothelium in development and disease. Front Physiol. (2014) 5:284. 10.3389/fphys.2014.00284 25136318 PMC4117979

[20] Mutsaers SE. Mesothelial cells: their structure, function and role in serosal repair. Respirology. (2002) 7:171–91. 10.1046/j.1440-1843.2002.00404.x 12153683

[21] Dixit R Ai X Fine A. Derivation of lung mesenchymal lineages from the fetal mesothelium requires hedgehog signaling for mesothelial cell entry. Development. (2013) 140:4398–406. 10.1242/dev.098079 24130328 PMC4007715

[22] Wilm B Muñoz-Chapuli R. The role of WT1 in embryonic development and normal organ homeostasis. In: Hastie N editor. The Wilms' Tumor (WT1) Gene: Methods and Protocols. New York, NY: Springer New York (2016). p. 23–39. 10.1007/978-1-4939-4023-3_3 27417957

[23] Que J Wilm B Hasegawa H Wang F Bader D Hogan BLM. Mesothelium contributes to vascular smooth muscle and mesenchyme during lung development. Proc Natl Acad Sci USA. (2008) 105:16626. 10.1073/pnas.0808649105 18922767 PMC2567908

[24] Cano E Carmona R Muñoz-Chápuli R. Wt1-expressing progenitors contribute to multiple tissues in the developing lung. Am J Physiol Lung Cell Mol Physiol. (2013) 305:L322–32. 10.1152/ajplung.00424.2012 23812634

[25] von Gise A Stevens SM Honor LB Oh JH Gao C Zhou B. Contribution of Fetal, but not adult, pulmonary mesothelium to mesenchymal lineages in lung homeostasis and fibrosis. Am J Respir Cell Mol Biol. (2016) 54:222–30. 10.1165/rcmb.2014-0461OC 26121126 PMC4821041

[26] Danopoulos S Shiosaki J Al Alam D. FGF signaling in lung development and disease: human versus mouse. Front Genet. (2019) 10:170. 10.3389/fgene.2019.00170 30930931 PMC6423913

[27] White AC Xu J Yin Y Smith C Schmid G Ornitz DM. FGF9 and SHH signaling coordinate lung growth and development through regulation of distinct mesenchymal domains. Development. (2006) 133:1507–17. 10.1242/dev.02313 16540513

[28] Yin Y Ornitz DM. FGF9 and FGF10 activate distinct signaling pathways to direct lung epithelial specification and branching. Sci Signal. (2020) 13(621). 10.1126/scisignal.aay4353 32127497 PMC7271816

[29] Yin Y Wang F Ornitz DM. Mesothelial- and epithelial-derived FGF9 have distinct functions in the regulation of lung development. Development. (2011) 138:3169–77. 10.1242/dev.065110 21750028 PMC3188607

[30] Takahashi H Friedmacher F Fujiwara N Hofmann A Puri P. Pulmonary FGF9 gene expression is downregulated during the pseudoglandular stage in nitrofen-induced hypoplastic lungs. Eur J Pediatr Surg. (2014) 24:75–8. 10.1055/s-0033-1351392 23897418

[31] van Loenhout RB Tseu I Fox EK Huang Z Tibboel D Post M. The pulmonary mesenchymal tissue layer is defective in an *in vitro* recombinant model of nitrofen-induced lung hypoplasia. Am J Pathol. (2012) 180:48–60. 10.1016/j.ajpath.2011.09.032 22063298

[32] Gilbert RM Schappell LE Gleghorn JP. Defective mesothelium and limited physical space are drivers of dysregulated lung development in a genetic model of congenital diaphragmatic hernia. Development. (2021) 148:dev199460. 10.1242/dev.199460 34015093 PMC8180258

[33] Kool H Mous D Tibboel D de Klein A Rottier RJ. Pulmonary vascular development goes awry in congenital lung abnormalities. Birth Defects Res C Embryo Today Rev. (2014) 102:343–58. 10.1002/bdrc.21085 25424472

[34] Varghese NP Tillman RH Keller RL. Pulmonary hypertension is an important co-morbidity in developmental lung diseases of infancy: bronchopulmonary dysplasia and congenital diaphragmatic hernia. Pediatr Pulmonol. (2021) 56:670–7. 10.1002/ppul.25258 33561308

[35] Mous DS Kool HM Wijnen R Tibboel D Rottier RJ. Pulmonary vascular development in congenital diaphragmatic hernia. Eur Respir Rev. (2018) 27:170104. 10.1183/16000617.0104-2017 29367409 PMC9488749

[36] Sluiter I van der Horst I van der Voorn P Boerema-de Munck A Buscop-van Kempen M de Krijger R. Premature differentiation of vascular smooth muscle cells in human congenital diaphragmatic hernia. Exp Mol Pathol. (2013) 94:195–202. 10.1016/j.yexmp.2012.09.010 23018129

[37] Yang MJ Russell KW Yoder BA Fenton SJ. Congenital diaphragmatic hernia: a narrative review of controversies in neonatal management. Transl Pediatr. (2021) 10:1432–47. 10.21037/tp-20-142 34189103 PMC8192986

[38] Acker SN Seedorf GJ Abman SH Nozik-Grayck E Kuhn K Partrick DA. Altered pulmonary artery endothelial–smooth muscle cell interactions in experimental congenital diaphragmatic hernia. Pediatr Res. (2015) 77:511–9. 10.1038/pr.2015.13 25580737 PMC4363155

[39] Kool HM Bürgisser PE Edel GG de Kleer I Boerema-de Munck A de Laat I. Inhibition of retinoic acid signaling induces aberrant pericyte coverage and differentiation resulting in vascular defects in congenital diaphragmatic hernia. Am J Physiol Lung Cell Mol Physiol. (2019) 317:L317–L31. 10.1152/ajplung.00104.2018 31268349

[40] Acker SN Seedorf GJ Abman SH Nozik-Grayck E Partrick DA Gien J. Pulmonary artery endothelial cell dysfunction and decreased populations of highly proliferative endothelial cells in experimental congenital diaphragmatic hernia. Am J Physiol Lung Cell Mol Physiol. (2013) 305:L943–L52. 10.1152/ajplung.00226.2013 24124189 PMC3882539

[41] Wakabayashi T Naito H Suehiro JI Lin Y Kawaji H Iba T. CD157 marks tissue-resident endothelial stem cells with homeostatic and regenerative properties. Cell Stem Cell. (2018) 22:384–97 e6. 10.1016/j.stem.2018.01.010 29429943

[42] Farkas S Simara P Rehakova D Veverkova L Koutna I. Endothelial progenitor cells produced from human pluripotent stem cells by a synergistic combination of cytokines, small compounds, and serum-free medium. Front Cell Dev Biol. (2020) 8:309. 10.3389/fcell.2020.00309 32509776 PMC7249886

[43] Bogenschutz EL Sefton EM Kardon G. Cell culture system to assay candidate genes and molecular pathways implicated in congenital diaphragmatic hernias. Dev Biol. (2020) 467:30–8. 10.1016/j.ydbio.2020.07.013 32827499 PMC7643881

[44] Kunisaki SM Jiang G Biancotti JC Ho KKY Dye BR Liu AP. Human induced pluripotent stem cell-derived lung organoids in an ex vivo model of the congenital diaphragmatic hernia fetal lung. STEM Cells Transl Med. (2021) 10:98–114. 10.1002/sctm.20-0199 32949227 PMC7780804

[45] Eenjes E van Riet S Kroon AA Slats AM Khedoe PPSJ Boerema-de Munck A. Disease modelling following organoid-based expansion of airway epithelial cells. Am J Physiol Lung Cell Mol Physiol. (2021) 321:L775–86. 10.1152/ajplung.00234.2020 34378410

[46] Hocking A Tommasi S Sordillo P Klebe S. The safety and exploration of the pharmacokinetics of intrapleural liposomal curcumin. Int J Nanomedicine. (2020) 15:943–52. 10.2147/IJN.S237536 32103948 PMC7023862

[47] Koopmans T Rinkevich Y. Mesothelial to mesenchyme transition as a major developmental and pathological player in trunk organs and their cavities. Commun Biol. (2018) 1:170. 10.1038/s42003-018-0180-x 30345394 PMC6191446

[48] Timo HL Carsten R Jennifer K Regine H Franziska G Irina W. Mesothelial mobilization in the developing lung and heart differs in timing, quantity, and pathway dependency. Am J Physiol Lung Cell Mol Physiol. (2019) 316:L767–83. 10.1152/ajplung.00212.2018 30702346

